# Acute and long-term effects of antibiotics commonly used in laboratory animal medicine on the fecal microbiota

**DOI:** 10.1186/s13567-020-00839-0

**Published:** 2020-09-14

**Authors:** Scott W. Korte, Rebecca A. Dorfmeyer, Craig L. Franklin, Aaron C. Ericsson

**Affiliations:** 1 Office of Animal Resources, University of Missouri, Columbia, MO USA; 2 Metagenomics Center, University of Missouri, Columbia, MO USA; 3 Mutant Mouse Resource and Research Center, University of Missouri, Columbia, MO USA; 4 Department of Veterinary Pathobiology, University of Missouri, Columbia, MO USA

## Abstract

Biomedical research relies on the use of animal models, and the animals used in those models receive medical care, including antibiotics for brief periods of time to treat conditions such as dermatitis, fight wounds, and suspected bacterial pathogens of unknown etiology. As many mouse model phenotypes are sensitive to changes in the gut microbiota, our goal was to examine the effect of antibiotics commonly administered to mice. Therefore, four treatment groups (subcutaneous enrofloxacin for 7 days, oral enrofloxacin for 14 days, oral trimethoprim-sulfamethoxazole for 14 days, and topical triple antibiotic ointment for 14 days) alongside a fifth control group receiving no treatment (*n* = 12/group) were included in our study. Fecal samples were collected prior to treatment, immediately after two weeks of exposure, and four weeks after cessation of treatment, and subjected to 16S rRNA library sequencing. The entire experimental design was replicated in mice from two different suppliers. As expected, several treatments including enrofloxacin and triple antibiotic ointment substantially decreased the amount of DNA recovered from fecal material, as well as the microbial richness. Notably, many of these effects were long-lasting with diminished gut microbiota (GM) richness four weeks following exposure, in both substrains of mice. Trimethoprim-sulfamethoxazole induced minimal to no discernible changes in the taxonomic composition beyond that seen in control mice. Collectively, these data highlight the need to consider the impact on GM of brief and seemingly routine use of antibiotics in the clinical care of research animals.

## Introduction

Comparative medicine is predicated on the use of animal models to provide a better understanding of the human condition, and mouse models in particular have become the backbone of biomedical research. When mouse models are used to study a disease process occurring in humans, a common practice is to strive for uniformity within the genetic background and environment of mice, by using inbred strains and providing a uniform diet, water, and housing to all mice. Moreover, all recognized infectious agents are excluded from many research colonies through the use of extensive sentinel screening, quarantine, and procedural programs. Despite all of these efforts however, the gut microbiota (GM) likely represents a significant source of residual biological variability and as such, has gained the attention of many researchers as a variable that should be acknowledged and considered in experimental design [1, 2]. The trillions of bacteria residing within the gut of our research animals (and ourselves) are now recognized as vital components of a healthy super-organism, and differences in specific characteristics of the GM of affected and unaffected individuals have been identified in most major medical conditions affecting humans. Using mouse models, causal relationships have been identified between many of these microbial features and disease severity, leading to an improved understanding of the pathogenesis and potentially insights into more tailored patient treatment and care.

The taxonomic composition of the GM of a given specific pathogen-free (SPF) mouse is determined by a multitude of factors but the maternal source is its starting point, and thus the facility wherein that mouse was born is likely the single greatest factor in its ultimate composition, as we and others have demonstrated substantial supplier-dependent differences in the GM of SPF mice [3, 4]. Once mice arrive at a new research institution, many other factors including shipping and acclimation [5], method of water purification [6], diet, bedding, and caging [7] can induce subtle or substantial changes in the composition of the GM at different levels of the gastrointestinal tract. At most institutions, laboratory mice are carefully monitored by technicians and laboratory animal veterinarians and brief administration of antibiotics is not uncommon to treat conditions ranging from C57BL/6 dermatitis and fight wounds to less justifiable causes such as colony-wide breeding slumps with no identifiable etiology. While the influence of chronic oral antibiotics on the GM is obvious and expectable, there is little information available regarding the effect of the two most commonly administered oral antibiotics for mice, trimethoprim-sulfamethoxazole (TMS) and enrofloxacin (Baytril®). Enrofloxacin can also be administered via intra-peritoneal (IP) injection, possibly providing a preferable route of administration with decreased direct exposure to, and influence on, the GM. Moreover, another commonly used antibiotic in mouse colonies is triple-antibiotic ointment (TAB) containing bacitracin, neomycin, and polymyxin B, with relatively broad activity against Gram-positive and Gram-negative organisms. While it is applied topically and usually for short durations, some portion is undoubtedly ingested during grooming and very little is known regarding its influence on the GM. Thus, we tested the influence of four different transient antibiotic treatments on the composition of the GM, sampling before antibiotic exposure, immediately after two weeks of exposure, and again four weeks after the discontinuation of treatment, with untreated mice serving as a control for time-dependent changes.

Considering the aforementioned differences in the GM composition of SPF mice from different suppliers, we were also interested to know whether antibiotic-mediated effects would be consistent across mice from different commercial sources. One might speculate that a richer GM might demonstrate increased resilience against the effects of certain antibiotics, as has been reported [8]. Therefore, all treatments were replicated in cohorts of C57BL/6J and C57BL/6NHsd mice purchased from the Jackson Laboratory and Envigo, respectively. In our experience, these suppliers generate mice with low and high richness GM relative to each other and mice at institutions across the biomedical research community [9]. Fecal DNA was used to generate 16S rRNA libraries which were sequenced on the Illumina MiSeq platform. DNA yields, detected richness, and antibiotic-induced changes were then assessed independently within each substrain (B6 or B6NHsd) relative to control mice of the same substrain.

## Methods

### Mice

Sixty six-week-old female C57BL/6NHsd (B6NHsd) mice and 60 six-week-old female C57BL/6J (B6J) mice were obtained from Envigo (Indianapolis, IN) and The Jackson Laboratory (Bar Harbor, ME), respectively. All animals were housed four per cage, by genotype, in individually ventilated cages (Thoren, Hazelton, PA) with compressed paper bedding (Paperchip® Brand Laboratory Animal Bedding, Shepherd Specialty Papers, Watertown, TN) and acidified, autoclaved water on a 14:10 h light/dark cycle. Mice received Purina LabDiet 5053 ad libitum. Cages were assembled with bedding, wire bar lids, and filter tops and autoclaved as a unit prior to use. All animal and cage manipulations were performed in a Class II A2 biosafety cabinet (LabGard® ES NU-540, Nuaire, Plymouth, MN 55447) disinfected with 10% bleach solution prior to use. All animals were housed in an AAALAC International-accredited facility and all animal use was performed according to the standards put forth in the Guide for the Care and Use of Laboratory Animals (8th ed.) and approved by the University of Missouri ACUC.

### Antibiotic treatment

Cages were randomly placed in one of five treatment groups, resulting in three cages of B6/Hsd and three cages of B6/J (4 mice/cage, n = 12/substrain) in each treatment group. Group 1 received trimethoprim-sulfamethoxazole (TMS) at a concentration of 0.5 mL/kg in the drinking water for 14 days; group 2 received enrofloxacin (Baytril®) at a concentration of 85 mg/kg in the drinking water for 14 days; group 3 received enrofloxacin via subcutaneous (IP) injections at 10 mg/kg, administered daily for 7 days; group 4 received topical triple antibiotic ointment containing neomycin, bacitracin, and polymyxin, a 0.1 g/mouse once daily for 14 days. Group 5, control mice, received no treatment, but were housed on the same rack, for the same duration as the other groups.

### Fecal sample collection

All sample collection occurred between 7 and 8 a.m., shortly after the beginning of the light cycle, within a biosafety cabinet. Fecal samples were collected one week after arrival at our institution and prior to any treatment (Pre), immediately after the full 7- or 14-day treatment period and 14 days later for the control group (Post), and four weeks after discontinuation of treatment or collection of the previous sample (4w post). To collect feces, mice were transferred individually from their home cage to an autoclaved cage containing no bedding or other material and allowed to defecate naturally. Following defecation, up to two fecal pellets per mouse were pierced with a sterile wooden toothpick and placed in a labeled, sterile 1.5 mL Eppendorf tube. Toothpicks were used once and discarded. Mice were then returned to their home cage and the collection cage wiped clean of any remaining fecal material or urine. Different collection cages were used for each treatment group and time-point. Following collection of all samples at a given time-point, samples were immediately transported to the laboratory and placed in a − 20 °C freezer until DNA extraction was performed.

### DNA extraction

Fecal DNA was extracted using an adaptation of the protocol published by Yu et al. [10]. Briefly, a single fecal pellet was placed in a 2 mL round-bottom tube containing 800 µL of lysis buffer (4% w/v sodium dodecyl sulfate, 500 mM NaCl, and 50 mM EDTA) and a single 0.5 cm-diameter stainless steel bead. Samples were then homogenized using a TissueLyser II (Qiagen, Venlo, the Netherlands) for three minutes at 30/sec, and then incubated at 70 °C for 20 min with periodic vortexing. Following centrifugation at 5000 × *g* for five minutes at room temperature, the supernatant was transferred to a sterile 1.5 mL Eppendorf tube. Each tube was then supplemented with 200 µL of 10 mM ammonium acetate, incubated on ice for five minutes, and then centrifuged as above. Up to 750 µL of the resultant supernatant was mixed with an equal volume of chilled isopropanol, incubated on ice for 30 min, and then centrifuged at 16,000 × *g* for 15 min at 4 °C. Following removal of the supernatant, the DNA pellet was washed and resuspended in 150 µL of Tris–EDTA. Following addition of 15 µL of proteinase-K and 200 µL of Buffer AL (DNeasy Blood and Tissue kit, Qiagen), samples were incubated at 70 °C for 10 min. Tubes were then supplemented with 200 µL of 100% ethanol, vortexed, transferred to a spin column from the DNeasy kit, and processed according to the manufacturer’s instructions and eluted in 200 µL of EB buffer (Qiagen). DNA yields were quantified via fluorometry (Qubit 2.0, Life Technologies, Carlsbad, CA) using quant-iT BR dsDNA reagent kits (Invitrogen, Carlsbad, CA).

### 16S rRNA library preparation and sequencing

Extracted fecal DNA was processed at the University of Missouri DNA Core Facility. Bacterial 16S rDNA amplicons were constructed via amplification of the V4 region of the 16 s rDNA gene with universal primers (U515F/806R) previously developed against the V4 region, flanked by Illumina standard adapter sequences [11, 12]. Oligonucleotide sequences are available at proBase [13]. A single forward primer and indexed reverse primers were used in all reactions. PCR was performed in 50 µL reactions containing 100 ng metagenomic DNA, primers (0.2 µM each), dNTPs (200 µM each), and Phusion high-fidelity DNA polymerase (1U). Amplification parameters were 98 °C^(3:00)^ + [98 °C^(0:15)^ + 50 °C^(0:30)^ + 72 °C^(0:30)^] × 25 cycles + 72 °C^(7:00)^. Amplicon pools (5 µL/reaction) were combined, thoroughly mixed, and then purified by addition of Axygen Axyprep MagPCR clean-up beads to an equal volume of 50 µL of amplicons and incubated for 15 min at room temperature. Products were then washed multiple times with 80% ethanol and the dried pellet was resuspended in 32.5 µL EB buffer, incubated for two minutes at room temperature, and then placed on the magnetic stand for five minutes. The final amplicon pool was evaluated using the Advanced Analytical Fragment Analyzer automated electrophoresis system, quantified using quant-iT HS dsDNA reagent kits, and diluted according to Illumina’s standard protocol for sequencing on the MiSeq instrument.

### Informatics analysis

Read merging, clustering, and annotation of DNA sequences was performed at the University of Missouri Informatics Research Core Facility. Paired DNA sequences were merged using FLASH software [14], and removed if found to be far from the expected length of 292 bases after trimming for base quality of 31. Cutadapt [15] (https://github.com/marcelm/cutadapt) was used to remove the primers at both ends of the contig and cull contigs that did not contain both primers. The research [16] fastq_filter command (https://drive5.com/usearch/manual/cmd_fastq_filter.html) was used for quality trimming of contigs, rejecting those for which the expected number of errors was greater than 0.5. All contigs were trimmed to 248 bases and shorter contigs were removed. The Qiime [17] v1.9 command split_libraries_fastq.py was used to demultiplex the samples. The outputs for all samples were combined into a single file for clustering. The uparse [18] method (https://www.drive5.com/uparse/) was used to both cluster contigs with 97% identity and remove chimeras. Taxonomy was assigned to selected OTUs using BLAST [19] against the SILVA database v128 [20] of 16S rRNA sequences and taxonomy.

### Statistical analysis

To identify substrain-dependent differences in initial bacterial richness (prior to treatment), non-normal data (as indicated via Shapiro–Wilk method) were compared via Mann–Whitney rank sum test. To identify time-dependent differences in bacterial richness, the numbers of distinct OTUs detected at each time-point within treatment groups were tested for normality and equal variance via the Shapiro–Wilk and Brown-Forsyth methods, respectively. Time-dependent main effects were then identified via one-way repeated measures ANOVA (normally distributed data with equal variance), Friedman repeated measures ANOVA on ranks (non-normally distributed data or data of unequal variance); post hoc pairwise comparisons were done using Bonferroni t-test respectively, all implemented in SigmaPlot 14.0. Differences in β-diversity were tested using permutational multivariate ANOVA implemented using Past software.

## Results

### Triple antibiotic ointment and enrofloxacin are associated with decreased fecal biomass

Review of the sequencing data generated from all 360 samples (2 mouse sources × 5 treatment groups × 3 time-points × 12 mice/group) revealed that seven samples failed to achieve a sufficient read count for use in downstream statistical analysis (range 0 to 107 reads/sample). Four of those seven failed samples were from a single treatment group and time-point (C57BL/6 J mice immediately after two weeks of treatment with triple antibiotic ointment), suggesting that the GM of this group was disproportionately affected by exposure to antibiotics. Comparison of DNA yields from a single fecal pellet, collected at roughly the same time of day for all groups, showed that samples from TAB-treated mice collected immediately following the exposure period provided the lowest DNA yields within both substrains, while treatment with enrofloxacin via either route of administration was associated with decreased DNA yields in one of the two mouse strains (Additional file 1). We interpreted these data to indicate that treatment with TAB or enrofloxacin (as described in “Methods”) is likely to transiently deplete the bacterial biomass within a typical fecal bolus. Of the remaining 353 samples, the mean (± SD) read count was 79,713 (± 21,599) reads, with a range from 22,808 to 139,595 reads per sample. To account for differential coverage between samples, data were randomly subsampled to a uniform depth of 22,807 reads. This rarefied dataset still resulted in Good’s coverage [21] of 0.996 (± 0.002), indicating that the subsampled data continue to capture the estimated true richness of the samples.

### Enrofloxacin and topical triple antibiotic ointment induce longstanding reduction in fecal richness

**Figure 1:**
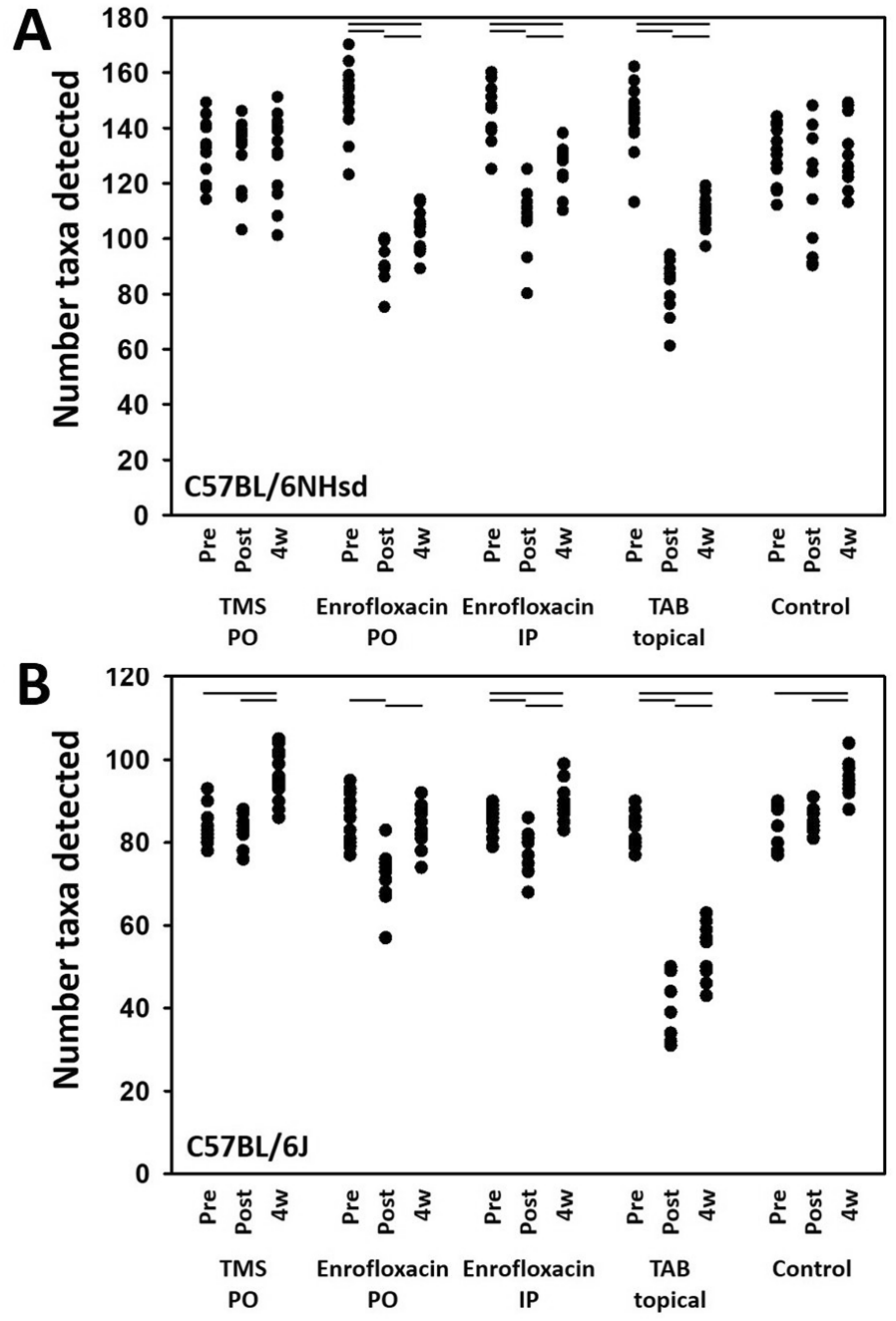
**Number of distinct OTUs detected in feces of adult C57BL/6NHsd (A) and C57BL/6 J (B) mice (n = 12/source) before (pre), immediately after (post), and four weeks after (4w) administration of trimethoprim-sulfamethoxazole (TMS); enrofloxacin (Baytril®) administered via two different routes; neomycin, bacitracin, polymyxin-B triple antibiotic (TAB); or no treatment (Control).** Lines indicate significant (*p* < 0.05) time-dependent differences within treatment group, as determined via one-way repeated measures ANOVA.

All studies were replicated in cohorts of mice from Envigo and The Jackson Laboratory to determine whether any treatment effects detected were consistent across different substrains harboring their own supplier-dependent GM profile. As our goal was to determine the degree, and duration, of the influence of antibiotics commonly used in the care of laboratory mice, all comparisons were stratified within each mouse substrain. In B6NHsd mice harboring a richer GM, treatment-dependent reductions in richness were detected in mice receiving enrofloxacin in the water or via injection, or topical TAB, and the richness remained significantly reduced 4 weeks later (Figure 1A). Similarly, in B6J mice colonized with a less rich GM, enrofloxacin via either route of administration and TAB were associated with significant reductions in richness immediately after two weeks of exposure (Figure 1B). Here however, fecal richness rebounded at 4 weeks post-treatment in mice receiving enrofloxacin, to a richness similar to, or even greater than, that prior to treatment. B6J mice receiving TAB maintained a significantly reduced fecal richness by 4 weeks post-treatment, similar to B6NHsd mice.

### Enrofloxacin and topical triple antibiotic ointment induce longstanding change in β-diversity

To assess the influence of the various treatments on overall community structure of the fecal microbiota, principal coordinate analysis (PCoA), using both unweighted (Jaccard) and weighted (Bray–Curtis) similarities, was performed for each treatment group. One-way permutational multivariate analysis of variance (PERMANOVA) using the same similarity metrics was used to confirm statistical significance and evaluate the source of any differences (i.e., differences in community membership or differences in the distribution of shared taxa).

**Figure 2:**
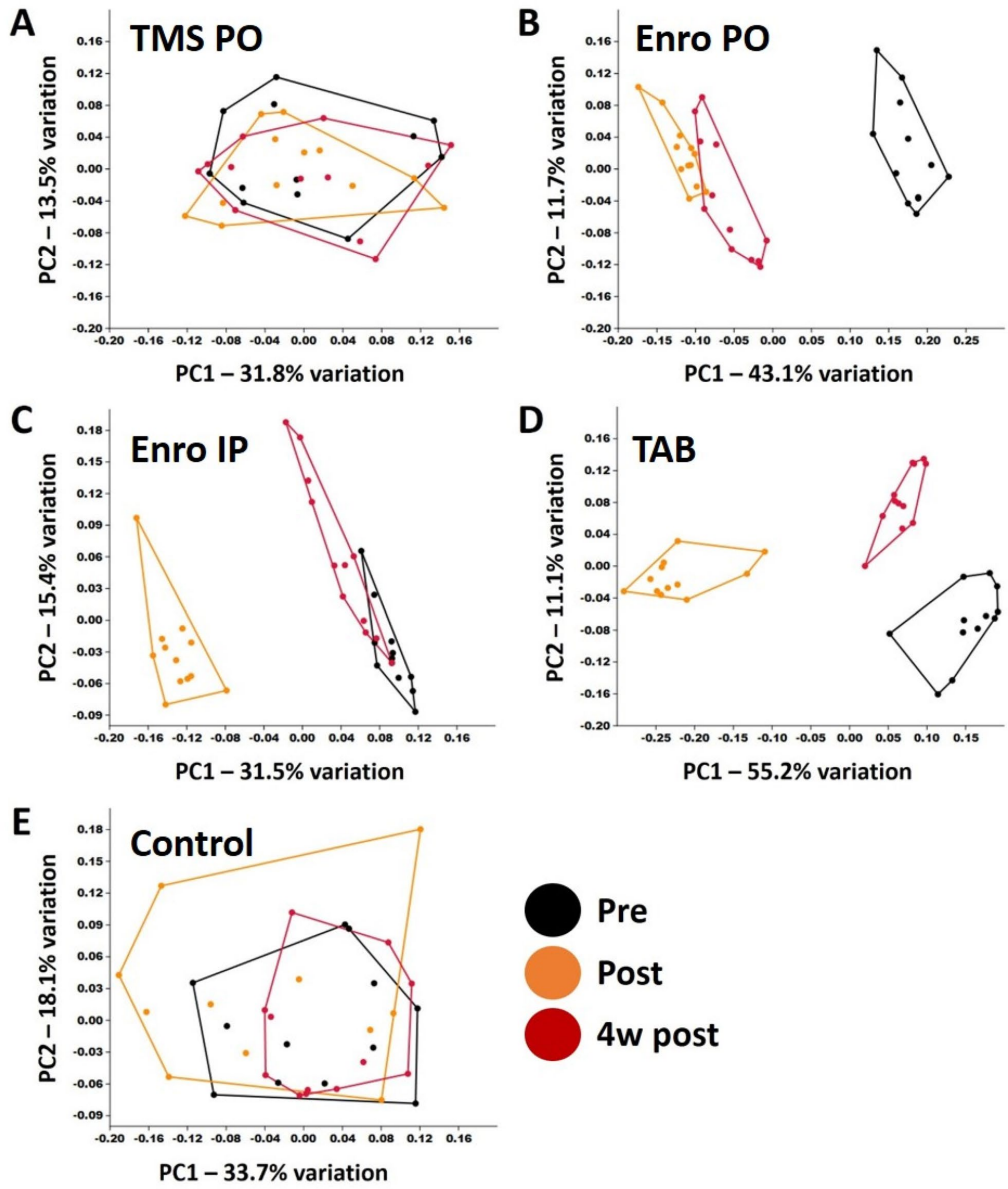
**Principal coordinate analysis of samples collected from C57BL/6NHsd mice(*n*** **= 12) before (pre), immediately after (post), and 4 weeks after (4w post) treatment with TMS PO (A), enrofloxacin PO (B), enrofloxacin IP (C), topical TAB (D), or no treatment (E), ordinated using Bray–Curtis similarity.**

PCoA plots generated from each separate B6NHsd treatment group revealed negligible change in the fecal bacterial communities of TMS-treated mice (or control mice) whereas the post-treatment samples from mice receiving enrofloxacin or TAB cluster separately from the pre-treatment samples (Figure 2). Notably, at four weeks post-treatment, the samples from mice receiving enrofloxacin PO or TAB were still distinctly separated from the pre-treatment samples, indicating those treatments induced a long-lasting compositional change in the GM. The same analyses, performed using a non-weighted similarity metric, showed a nearly identical pattern of treatment effects (Additional file 2). Ordination of all treatment groups collectively provides a visual comparison of the relative effect size of each treatment (Additional file 3). One-way PERMANOVA testing confirmed significant main effects of enrofloxacin PO and IP and TAB, regardless of the similarity metric used (Table 1). Pairwise comparisons between pre-treatment samples and either of the post-treatment time-points yielded similar results in all tests. No significant time-dependent differences were detected in TMS-treated mice. There was a significant main effect of time in the control group of B6NHsd mice when testing was performed using the non-weighted Jaccard similarity (*p* = 0.0397). Collectively however, the modest F value associated with that test (F = 1.81), the lack of significant differences in pairwise comparisons, and the substantial overlap in the PCoA plots suggests that this main effect is minimal and likely reflects normal random variability over time.

**Table 1:** **Results of one-way PERMANOVA to detect differences in fecal community structure between pre-treatment (Pre), immediately post-treatment (Post), and four weeks post-treatment (4w post) samples collected from C57BL/6NHsd mice receiving the indicated treatments (** ***n*** **= 12)**

Treatment	Similarity	Main effects		Pre vs post		Pre vs 4w post	
		*p*	F	*p*	F	*p*	F
TMS	Bray–Curtis	0.34	1.06	0.26	1.25	0.31	1.06
	Jaccard	0.07	1.53	0.11	1.52	0.05	1.90
Enrofloxacin PO	Bray–Curtis	0.002	4.11	0.001	5.86	0.006	4.51
	Jaccard	0.0001	15.95	0.0001	23.71	0.0001	20.56
Enrofloxacin IP	Bray–Curtis	0.004	3.36	0.01	4.03	0.07	2.40
	Jaccard	0.0001	11.97	0.0001	16.69	0.0001	6.09
TAB	Bray–Curtis	0.0001	27.74	0.0001	40.20	0.0005	7.16
	Jaccard	0.0001	20.58	0.0001	29.25	0.0001	14.23
Control	Bray–Curtis	0.35	1.06	0.44	0.87	0.30	1.09
	Jaccard	0.04	1.81	0.08	1.84	0.15	1.39

**Figure 3:**
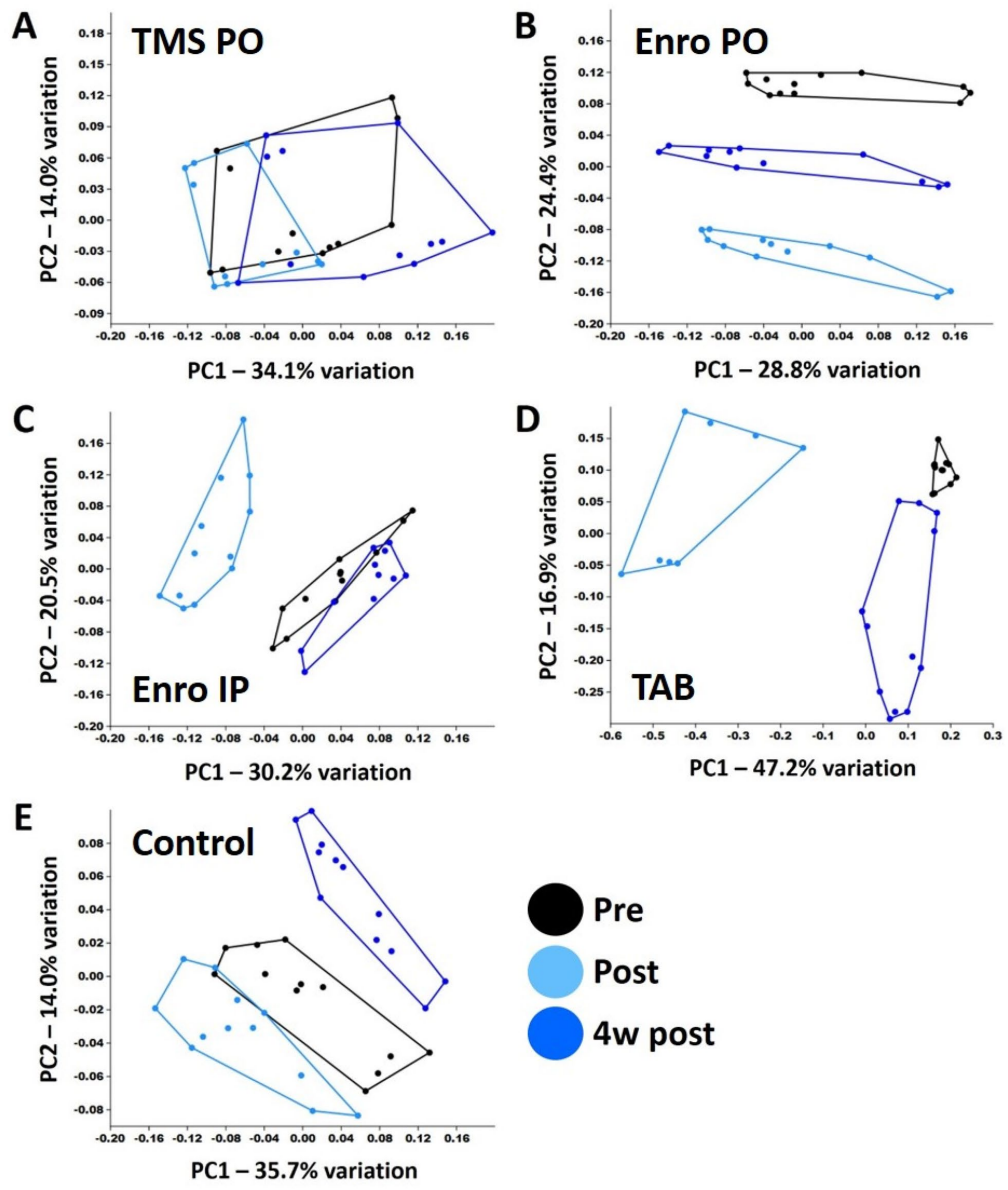
**Principal coordinate analysis of samples collected from C57BL/6 J mice (*n***  **= 12) before (pre), immediately after (post), and 4 weeks after (4w post) treatment with TMS PO (A), enrofloxacin PO (B), enrofloxacin IP (C), topical TAB (D), or no treatment (E), ordinated using Bray–Curtis similarity.**

An analysis of B6J mice demonstrated similar effects of treatment, albeit with a few notable exceptions. As with samples from B6NHsd mice, PCoA revealed clear time-dependent separation of samples in groups receiving enrofloxacin (PO or IP) and TAB, and no separation by time-point in mice receiving TMS based on weighted similarities (Figure 3). In contrast to B6NHsd mice however, a clear separation of pre-, post-, and four weeks post-treatment samples in control mice was observed, indicating the fecal community in this group was also undergoing subtle changes over time. PERMANOVA tests performed using Bray–Curtis similarity detected significant main effects of time in all groups except mice receiving enrofloxacin IP. However, the *p* and pseudo-F values associated with those tests were relatively modest, the exception being samples from TAB-treated mice (Table 2). When ordination and testing of B6J samples was performed using an unweighted similarity metric (Additional file 4, Table 2), significant main effects were detected in all treatment groups, although pairwise comparisons suggested that the acute changes in GM composition (i.e., from pre- to post-treatment) were most substantial in enrofloxacin- and TAB-treated mice. Similarly, while comparison of pre-treatment samples to those collected four weeks post-treatment yielded *p* values below 0.05 in all groups, the pseudo-F value associated with those results was greater in TAB-treated mice than in the control group, whereas testing of the other groups resulted in *p* and F values comparable to those generated in untreated control mice. Collectively, these data suggest that the GM of B6J mice treated with TAB was particularly affected by treatment. Again, ordination of all treatment groups collectively provides a visual comparison of the relative effect size of each treatment (Additional file 5).

**Table 2:** **Results of one-way PERMANOVA to detect differences in fecal community structure between pre-treatment (Pre), immediately post-treatment (Post), and four weeks post-treatment (4w post) samples collected from C57BL/6 J mice receiving the indicated treatments (*n***  **= 12)**

Treatment	Similarity	Main effects		Pre vs post		Pre vs 4w post	
		*p*	F	*p*	F	*p*	F
TMS	Bray–Curtis	0.04	3.15	0.11	2.49	0.25	1.35
	Jaccard	0.0001	4.90	0.02	2.02	0.0001	5.67
Enrofloxacin PO	Bray–Curtis	0.0313	2.89	0.03	3.76	0.16	1.80
	Jaccard	0.0001	6.81	0.0001	11.12	0.0001	5.48
Enrofloxacin IP	Bray–Curtis	0.1334	1.80	0.20	1.61	0.45	0.75
	Jaccard	0.0001	8.01	0.0001	7.32	0.0001	5.68
TAB	Bray–Curtis	0.0001	9.58	0.0001	13.01	0.0007	11.56
	Jaccard	0.0001	11.31	0.0001	15.83	0.0001	14.70
Control	Bray–Curtis	0.0122	3.94	0.052	3.54	0.28	1.22
	Jaccard	0.0001	6.34	0.0031	2.44	0.0001	6.67

To provide a fine-toothed profile of the bacterial taxa affected by each treatment, serial Kruskal–Wallis ANOVA on ranks was performed on all detected taxa (within each treatment group), with *p* values adjusted to control the FDR associated with multiple tests. Additional files 6, 7, 8, 9, 10, 11, 12, 13, 14 list the taxonomies found to differ between time-points in each group, alongside the *p* value, timing, and directionality associated with the detected difference. While an exhaustive description of those differences is not possible, several general observations were made. First, the majority of detected differences were, as expected, decreases in the relative abundance of specific taxa from a wide range of bacterial families, particularly in samples from mice receiving enrofloxacin (via either route) or TAB. Second, while many of those reductions, or even eliminations, of taxa post-treatment were transient, many were sustained at four weeks post-treatment, particularly in mice receiving enrofloxacin PO or TAB. Third, comparison of the effects of enrofloxacin via the two routes of administration indicated a greater and more prolonged effect of PO versus IP administration within both substrains. Lastly, a careful comparison of the taxa found to increase following each of the different antibiotic exposures found several commonalities including one or more lactic acid bacteria (e.g., *Enterococcus* sp., *Lactobacillus* sp., and unidentified *Lactobacillales*) following exposure to all of the antibiotics, *Desulfovibrio* sp. following exposure to TAB, and *Akkermansia* sp. following exposure to several antibiotics.

**Figure 4:**
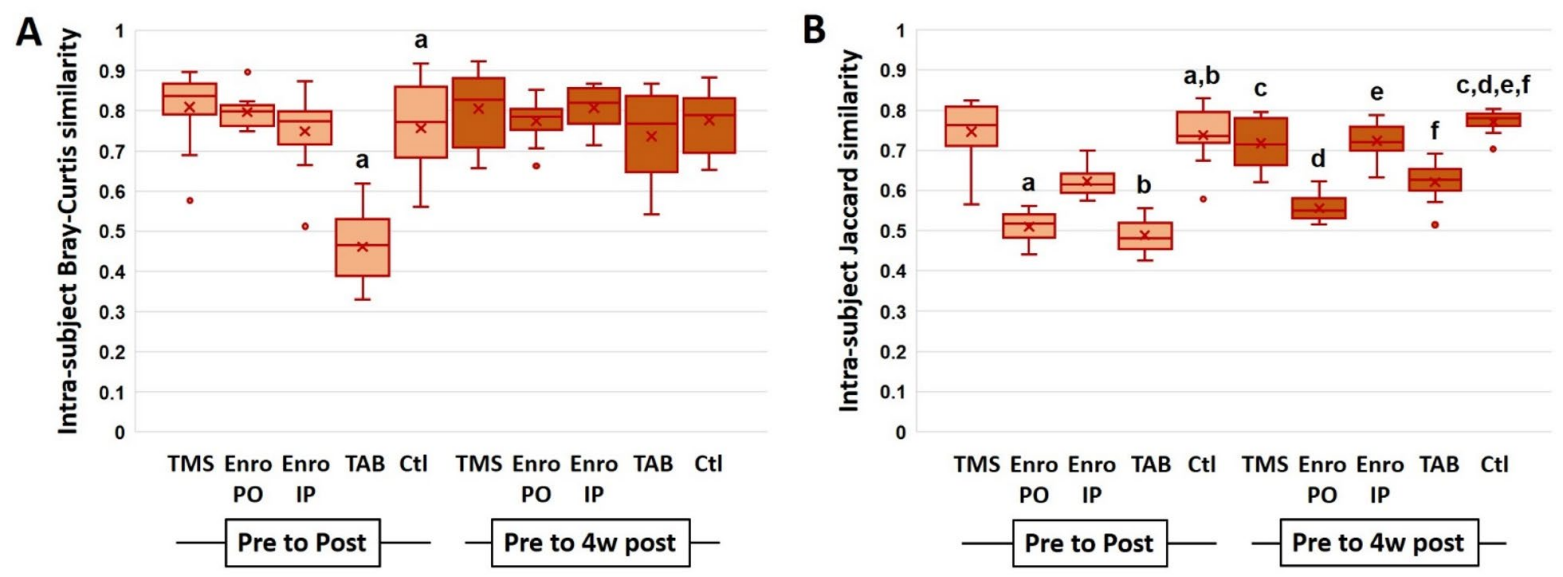
**Intra-individual Bray–Curtis (A) and Jaccard (B) similarity between pre- and post-treatment (light boxes), and pre- and four weeks post-treatment (dark boxes), fecal microbiota of C57BL/6NHsd mice. Like letters indicate significant reduction relative to the differences observed in control mice.**

**Figure 5:**
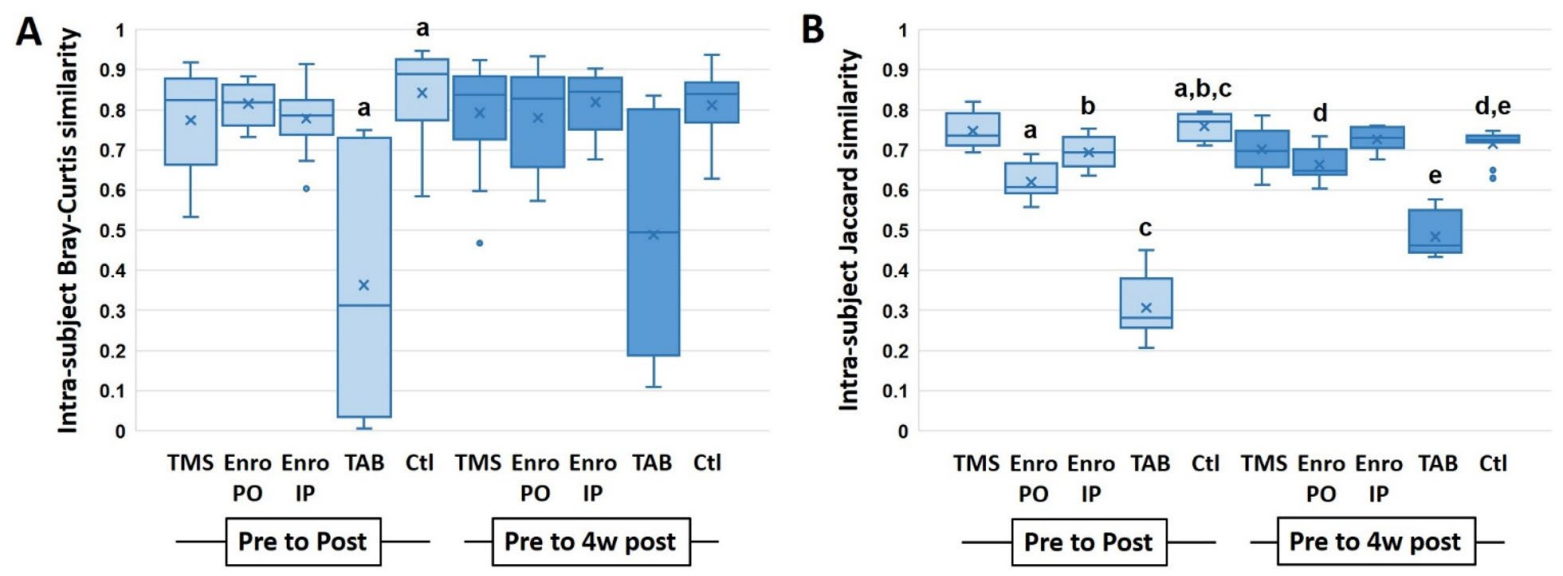
**Intra-individual Bray–Curtis (A) and Jaccard (B) similarity between pre- and post-treatment (light boxes), and pre- and four weeks post-treatment (dark boxes), fecal microbiota of C57BL/6J mice. Like letters indicate significant reduction relative to the differences observed in control mice.**

In order to control for inter-individual variation, intra-individual Bray–Curtis and Jaccard similarities between baseline and the later time-points were determined for all groups. Within the B6NHsd substrain, only TAB resulted in a significantly greater change from baseline to post-treatment than that seen in untreated control mice (Figure 4A). By four weeks post-treatment however, intra-individual change assessed via this similarity metric was similar in all groups (Figure 4A). When change over time was determined using Jaccard similarities (based on shared presence or absence of taxa, regardless of relative abundance), mice exposed to enrofloxacin PO or topical TAB both demonstrated a significantly reduced similarity between baseline and post-treatment samples relative to control mice, and all four experimental groups showed a significantly reduced intra-individual Jaccard similarity between baseline and four weeks post-treatment (Figure 4B). Analysis of the B6J substrain revealed a very similar pattern including a significant reduction in intra-individual Bray–Curtis similarity from baseline to post-treatment in mice receiving TAB (Figure 5A). As in B6NHsd, this difference was no longer present at four weeks post-treatment (despite a trend toward continued reduction). Comparison of intra-individual Jaccard similarities detected significant reductions in mice receiving enrofloxacin via either route or TAB at the post-treatment time-point, and in all groups at four weeks post-treatment, relative to control mice (Figure 5B).

In summary, the data presented above demonstrate that many of the antibiotic regimens used in the field of clinical laboratory animal medicine, both prophylactically and therapeutically, induce profound and possibly longstanding changes in the gut microbiota of mice. Supporting the broad applicability of these data, the observed effects were consistent in B6 substrains from suppliers with distinct GM. In particular, enrofloxacin and topical TAB demonstrated the greatest influence in terms of community structure and complete loss of detectable taxa, while oral TMS was associated with negligible change beyond that seen in control mice. These findings highlight the need to carefully record and consider the use of such medications on animals currently involved in research, whether the GM is a measurable outcome or not.

## Discussion

These studies add to the emerging realization that antibiotics used transiently to treat clinical signs or induce gene transcription (e.g., doxycycline-induced Tet-on systems) can have profound and long-lasting effects on the GM [22, 23], and potentially, the model phenotype. With this in mind, mouse models that are sensitive to the composition of the GM [24−26] are at risk of being influenced by the presence of antibiotic exposures during the study. As the treatments selected for testing are all commonly administered antibiotics for laboratory mice, there is the chance that certain treatments may be administered by laboratory animal care staff unbeknownst to the investigator. Such treatments thus represent potential confounds in models the phenotype of which are modulated by the GM. Of particular note in this regard is triple antibiotic ointment, a mainstay in rodent vivaria for the treatment of B6 dermatitis, fight wounds, and other conditions, and often applied without direct veterinary supervision.

Supporting the broad applicability of these data, similar effects were observed in mice of two substrains, harboring GM of relatively low and high richness. Specifically, oral TMS induced negligible effect on the microbiota in either substrain, and enrofloxacin and TAB induced the greatest changes in richness and β-diversity in both substrains. Recent findings have demonstrated a certain resistance, in the general sense, to antibiotic perturbation in mice harboring GM derived from wild mice [8], and we hypothesized the richer microbiota typically found in mice from Envigo would exhibit a similar effect. While the proportionate reduction in richness seen in B6NHsd mice was comparable or greater than in B6J for the three treatments with an observed effect, the nadir in richness observed in B6NHsd mice was much higher than in B6J mice due to the difference in starting richness. Collectively, these data suggest that both GMs are similarly affected by the antibiotics tested.

While the effect of orally administered antibiotics on the GM may be intuitive, there were several notable findings in the current study. First, these data document a significant acute influence of enrofloxacin on the GM regardless of the route of administration, although the effect of oral administration was more prolonged relative to IP administration. Enrofloxacin is excreted via hepatobiliary and renal mechanisms, but the biliary fraction undergoes enterohepatic recirculation and high concentrations are achieved in the intestinal lumen, even following systemic administration [27, 28]. Enrofloxacin is bacteriostatic at lower concentrations and bactericidal at higher concentrations, and the present data suggest that both routes of administration resulted in sufficient levels within the gut lumen to achieve bactericidal properties. For antibiotics eliminated via the feces such as enrofloxacin, coprophagy may also serve to amplify or perpetuate the effects of the compound. These data suggest that, if enrofloxacin is to be used in a potentially sensitive model, IP administration may be preferable to PO although both can be expected to induce some degree of change in the GM.

TMS is bactericidal in most situations owing to the synergistic effects of trimethoprim when combined with a sulfonamide. However, orally administered TMS is absorbed in the upper GI tract, distributes to tissues, and is largely eliminated through renal excretion in the urine [29, 30]. The fraction that is not eliminated in urine is metabolized in the liver to inactive compounds [31]. This may explain, at least partially, the relative sparing of the gut microbiota in mice receiving oral TMS. It should be noted though, that the lack of discernible effect on the fecal microbiota does not obviate the possibility of effects on biologically relevant microbes in the upper GI microbiota, subtle effects on the fecal microbiota below the limit of detection, or non-antimicrobial effects on the host, capable of altering a model phenotype. As a relevant example, studies using a transgenic mouse model of Devic’s disease experienced a complete loss of a robust phenotype following exposure to TMS in the drinking water, and disease was restored following co-housing with wild-type mice unexposed to antibiotics, strongly suggesting a microbiota-dependent phenotype that was interrupted by oral TMS [32].

It should also be noted that there are several possible confounding factors introduced by the different routes of administration, including differences in the amount of handling required for IP or topical administration of medications as compared to administration through the drinking water, or the potential for decreased water consumption in groups receiving treatments in the drinking water. As the ultimate goal of these studies was to determine the influence of antibiotic regimens used in laboratory animal medicine, we opted to perform all treatments exactly as they would be done in clinical settings, without controlling for the amount of handling or water consumption. Thus, the effects observed in each treatment group represent the totality of all influences associated with that treatment, and not necessarily the antibiotic treatment, per se.

Ultimately, the influence of antibiotics on the phenotype of a given rodent model may depend on a multitude of factors including duration of exposure, formulation, acidification of the rodent drinking water (a common practice to reduce bacterial growth), and even host genetics. Researchers and laboratory animal veterinarians needing to treat research mice, either as individuals or at the colony level, should consider initially treating a subset of animals if possible to determine effects on the model phenotype. Additionally, several fecal samples should be collected from mice prior to administration of antibiotics. These may be useful for comparison to post-treatment samples should a change in phenotype occur, or even a source for reconstitution of the GM following cessation of treatment.

## Supplementary information

**Additional file 1.** DNA yields from a single fecal pellet of adult C57BL/6NHsd (**A**) and C57BL/6J (**B**) mice (n = 12/source) before (pre), immediately after (post), and four weeks after (4w) administration of trimethoprim-sulfamethoxazole (TMS); enrofloxacin (Baytril®) administered via two different routes; neomycin, bacitracin, polymyxin-B triple antibiotic (TAB); or no treatment (Control). Lines indicate significant (p < 0.05) time-dependent differences within treatment group, as determined via one-way repeated measures ANOVA.

**Additional file 2.** Principal coordinate analysis of samples collected from C57BL/6NHsd mice (n = 12) before (pre), immediately after (post), and 4 weeks after (4w post) treatment with TMS PO (**A**), enrofloxacin PO (**B**), enrofloxacin IP (**C**), topical TAB (**D**), or no treatment (**E**), ordinated using Jaccard similarity.

**Additional file 3.** Principal coordinate analysis of samples collected from C57BL/6NHsd mice (n = 12) before (pre), immediately after (post), and 4 weeks after (4w post) treatment with TMS PO, enrofloxacin PO, enrofloxacin IP, topical triple antibiotic (TAB), or no treatment (Control), ordinated using Bray-Curtis (**A**) or Jaccard (**B**) similarities.

**Additional file 4.** Principal coordinate analysis of samples collected from C57BL/6J mice (n = 12) before (pre), immediately after (post), and 4 weeks after (4w post) treatment with TMS PO (**A**), enrofloxacin PO (**B**), enrofloxacin IP (**C**), topical TAB (**D**), or no treatment (**E**), ordinated using Jaccard similarity.

**Additional file 5.** Principal coordinate analysis of samples collected from C57BL/6J mice (n = 12) before (pre), immediately after (post), and 4 weeks after (4w post) treatment with TMS PO, enrofloxacin PO, enrofloxacin IP, topical triple antibiotic (TAB), or no treatment (Control), ordinated using Bray-Curtis (**A**) or Jaccard (**B**) similarities.

**Additional file 6.** OTUs found to differ between baseline and later timepoints in C57BL/6NHsd mice following exposure to TMS PO. Phylum, family, and best taxonomic resolution (Taxonomy) are provided, along with the FDR-adjusted p value, and the relative abundance of that taxa relative to baseline immediately post-treatment (post) and four weeks post-treatment (4w).

**Additional file 7.** OTUs found to differ between baseline and later timepoints in C57BL/6NHsd mice following exposure to enrofloxacin PO. Phylum, family, and best taxonomic resolution (Taxonomy) are provided, along with the FDR-adjusted p value, and the relative abundance of that taxa relative to baseline immediately post-treatment (post) and four weeks post-treatment (4w).

**Additional file 8.** OTUs found to differ between baseline and later timepoints in C57BL/6NHsd mice following exposure to enrofloxacin IP. Phylum, family, and best taxonomic resolution (Taxonomy) are provided, along with the FDR-adjusted p value, and the relative abundance of that taxa relative to baseline immediately post-treatment (post) and four weeks post-treatment (4w).

**Additional file 9.** OTUs found to differ between baseline and later timepoints in C57BL/6NHsd mice following exposure to topical TAB. Phylum, family, and best taxonomic resolution (Taxonomy) are provided, along with the FDR-adjusted p value, and the relative abundance of that taxa relative to baseline immediately post-treatment (post) and four weeks post-treatment (4w).

**Additional file 10.** OTUs found to differ between baseline and later timepoints in C57BL/6J mice following exposure to TMS PO. Phylum, family, and best taxonomic resolution (Taxonomy) are provided, along with the FDR-adjusted p value, and the relative abundance of that taxa relative to baseline immediately post-treatment (post) and four weeks post-treatment (4w).

**Additional file 11.** OTUs found to differ between baseline and later timepoints in C57BL/6J mice following exposure to enrofloxacin PO. Phylum, family, and best taxonomic resolution (Taxonomy) are provided, along with the FDR-adjusted p value, and the relative abundance of that taxa relative to baseline immediately post-treatment (post) and four weeks post-treatment (4w).

**Additional file 12.** OTUs found to differ between baseline and later timepoints in C57BL/6J mice following exposure to enrofloxacin IP. Phylum, family, and best taxonomic resolution (Taxonomy) are provided, along with the FDR-adjusted p value, and the relative abundance of that taxa relative to baseline immediately post-treatment (post) and four weeks post-treatment (4w).

**Additional file 13.** OTUs found to differ between baseline and later timepoints in C57BL/6J mice following exposure to topical TAB. Phylum, family, and best taxonomic resolution (Taxonomy) are provided, along with the FDR-adjusted p value, and the relative abundance of that taxa relative to baseline immediately post-treatment (post) and four weeks post-treatment (4w).

**Additional file 14.** OTUs found to differ between baseline and later timepoints in untreated control C57BL/6J mice. Phylum, family, and best taxonomic resolution (Taxonomy) are provided, along with the FDR-adjusted p value, and the relative abundance of that taxa relative to baseline immediately post-treatment (post) and four weeks post-treatment (4w).

## Data Availability

All 16S rRNA amplicon sequencing data have been uploaded to the National Center for Biotechnology Information (NCBI) Sequence Read Archive (SRA) and are available as BioProject PRJNA629378.

## Abbreviations

B6J: C57BL/6J
B6NHsd: C57BL/6NHsd
GM: gut microbiota
IP: intra-peritoneal
OTU: operational taxonomic unit
PCoA: principal coordinate analysis
PO: per os (Oral)
SPF: specific pathogen-free
TAB: triple antibiotic ointment
TMS: trimethoprim-sulfamethoxazole
