# Supplementary figures and images for: Acute and long-term effects of antibiotics commonly used in laboratory animal medicine on the fecal microbiota
Source: Vet Res. 2020 Sep 14;51:116. doi: 10.1186/s13567-020-00839-0 (PMC7489021; doi:10.1186/s13567-020-00839-0)

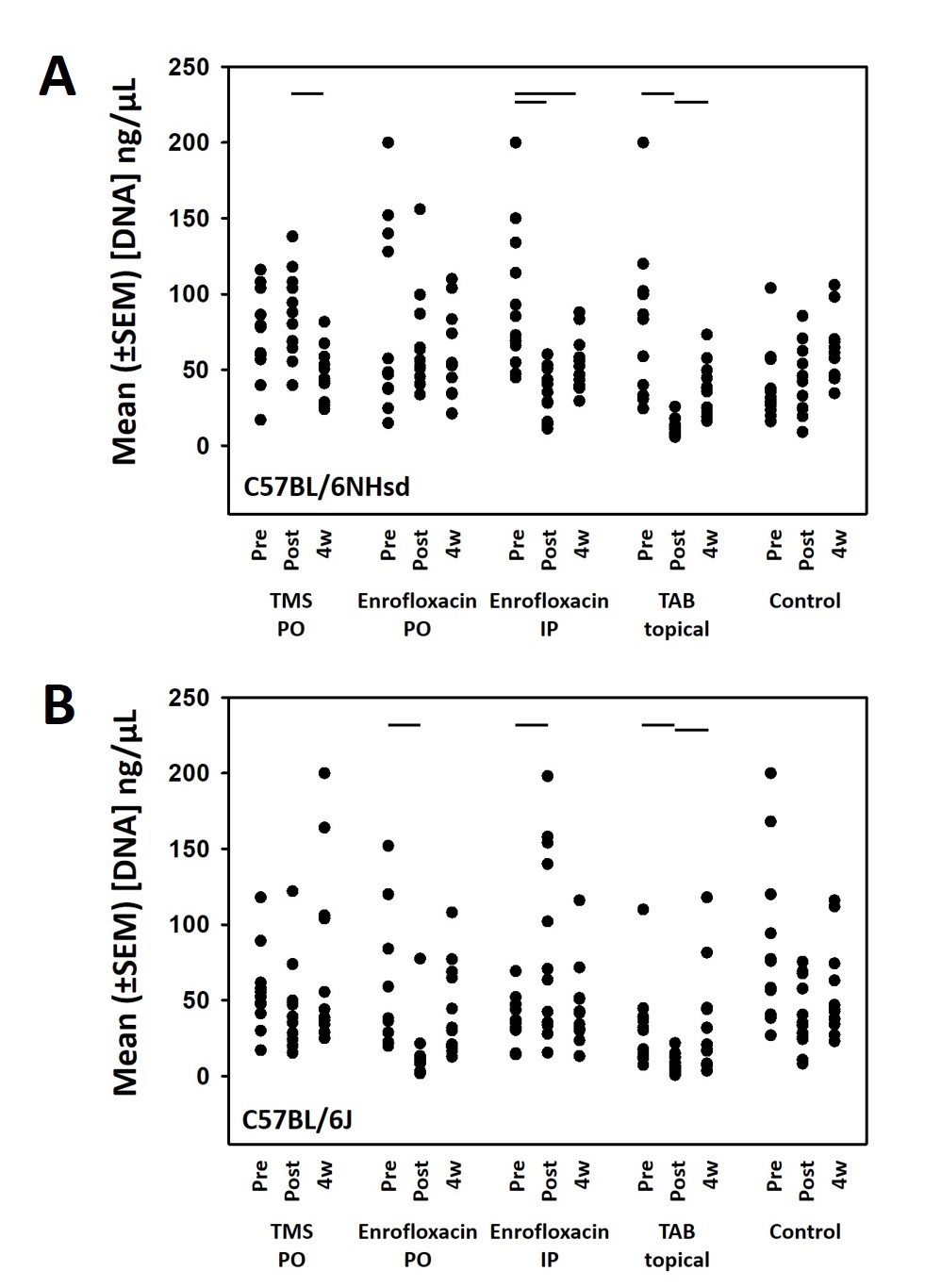

Supplement: Supplementary file 1 — Additional file 1. DNA yields from a single fecal pellet of adult C57BL/6NHsd (A) and C57BL/6J (B) mice (n = 12/source) before (pre), immediately after (post), and four weeks after (4w) administration of trimethoprim-sulfamethoxazole (TMS); enrofloxacin (Baytril®) administered via two different routes; neomycin, bacitracin, polymyxin-B triple antibiotic (TAB); or no treatment (Control). Lines indicate significant (p < 0.05) time-dependent differences within treatment group, as determined via one-way repeated measures ANOVA. [file 13567_2020_839_MOESM1_ESM.jpg]

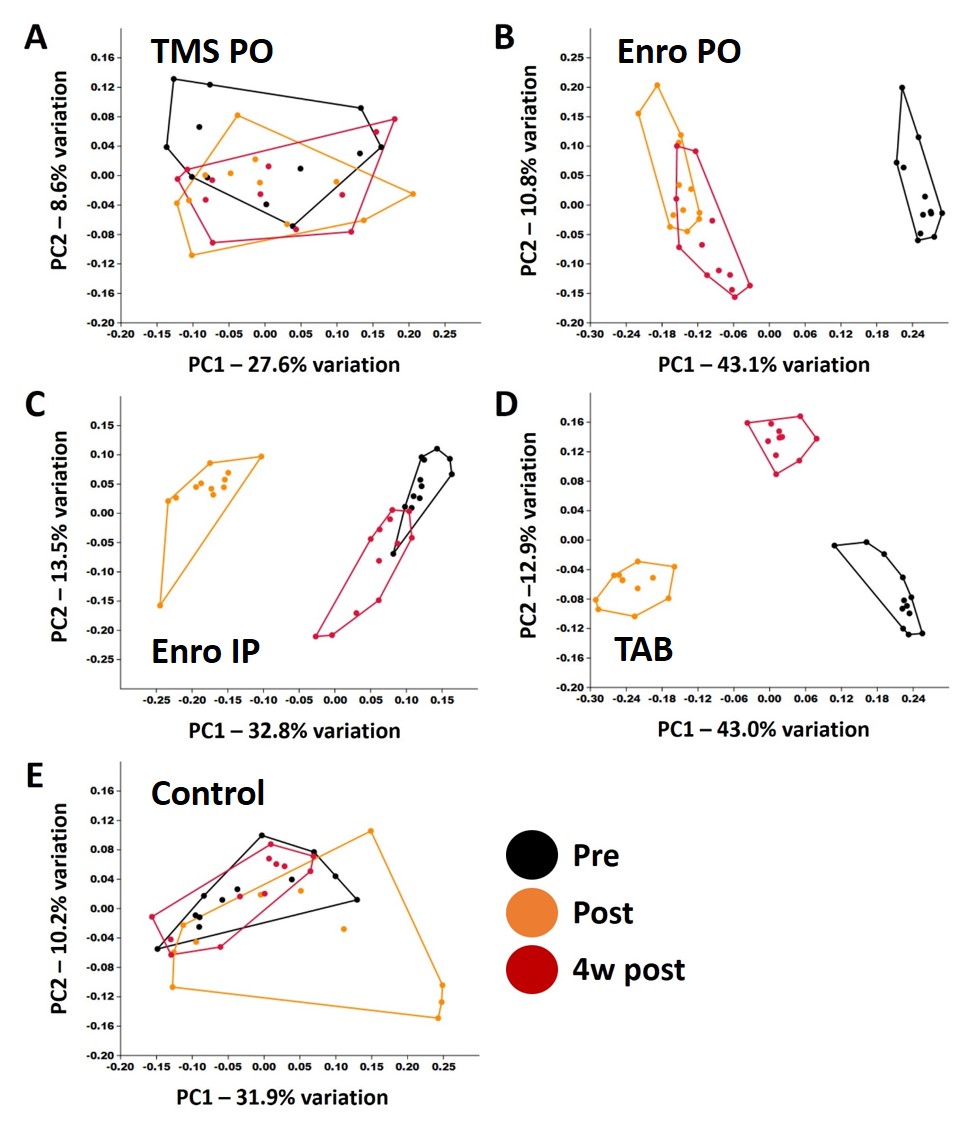

Supplement: Supplementary file 2 — Additional file 2. Principal coordinate analysis of samples collected from C57BL/6NHsd mice (n = 12) before (pre), immediately after (post), and 4 weeks after (4w post) treatment with TMS PO (A), enrofloxacin PO (B), enrofloxacin IP (C), topical TAB (D), or no treatment (E), ordinated using Jaccard similarity. [file 13567_2020_839_MOESM2_ESM.jpg]

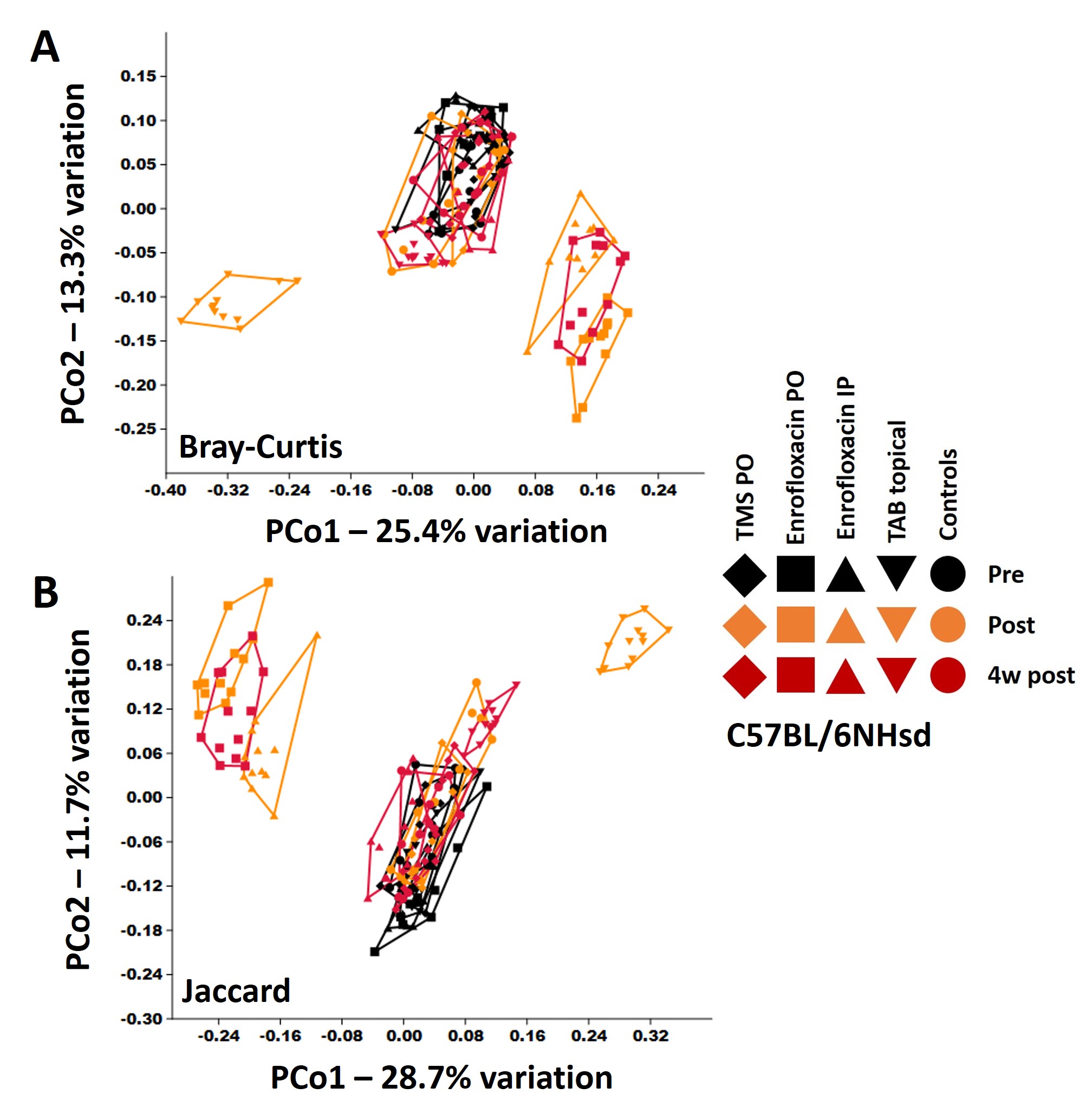

Supplement: Supplementary file 3 — Additional file 3. Principal coordinate analysis of samples collected from C57BL/6NHsd mice (n = 12) before (pre), immediately after (post), and 4 weeks after (4w post) treatment with TMS PO, enrofloxacin PO, enrofloxacin IP, topical triple antibiotic (TAB), or no treatment (Control), ordinated using Bray-Curtis (A) or Jaccard (B) similarities. [file 13567_2020_839_MOESM3_ESM.jpg]

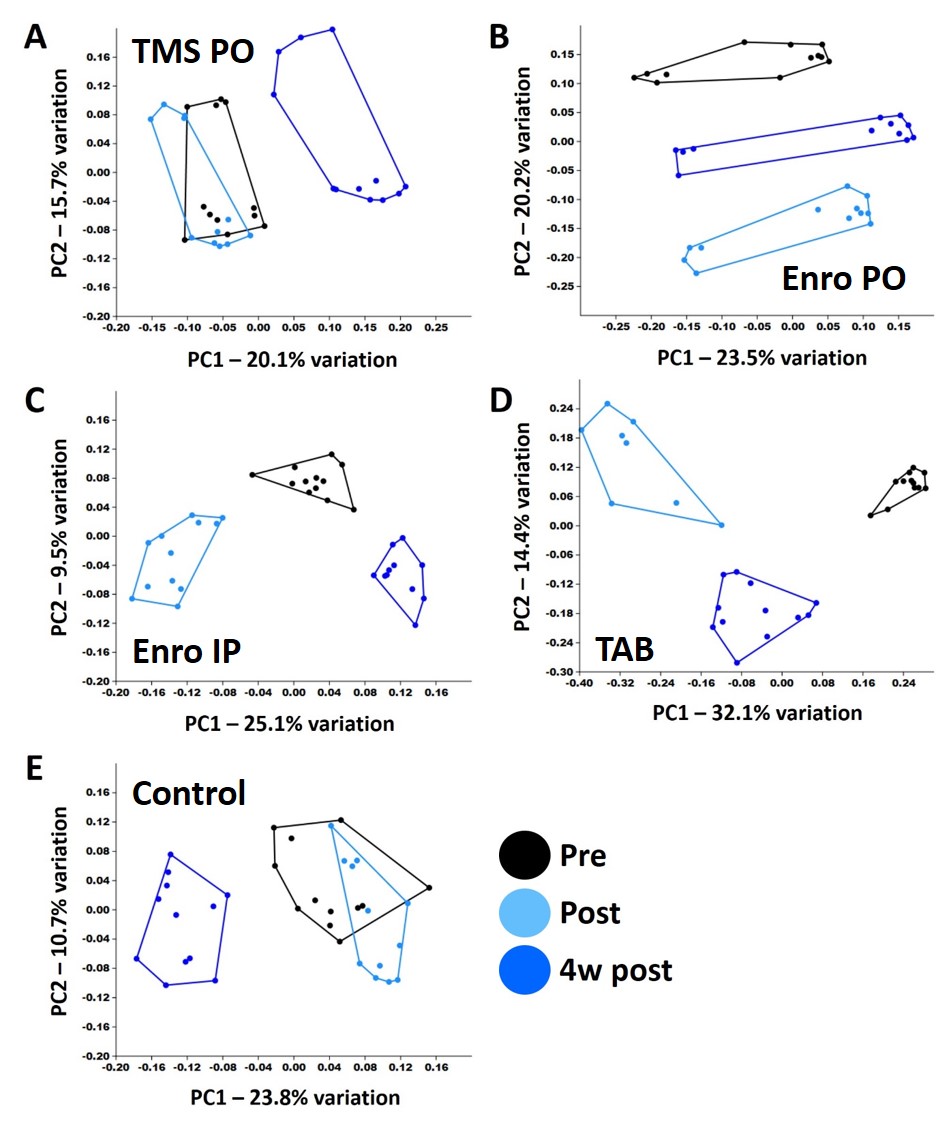

Supplement: Supplementary file 4 — Additional file 4. Principal coordinate analysis of samples collected from C57BL/6J mice (n = 12) before (pre), immediately after (post), and 4 weeks after (4w post) treatment with TMS PO (A), enrofloxacin PO (B), enrofloxacin IP (C), topical TAB (D), or no treatment (E), ordinated using Jaccard similarity. [file 13567_2020_839_MOESM4_ESM.jpg]

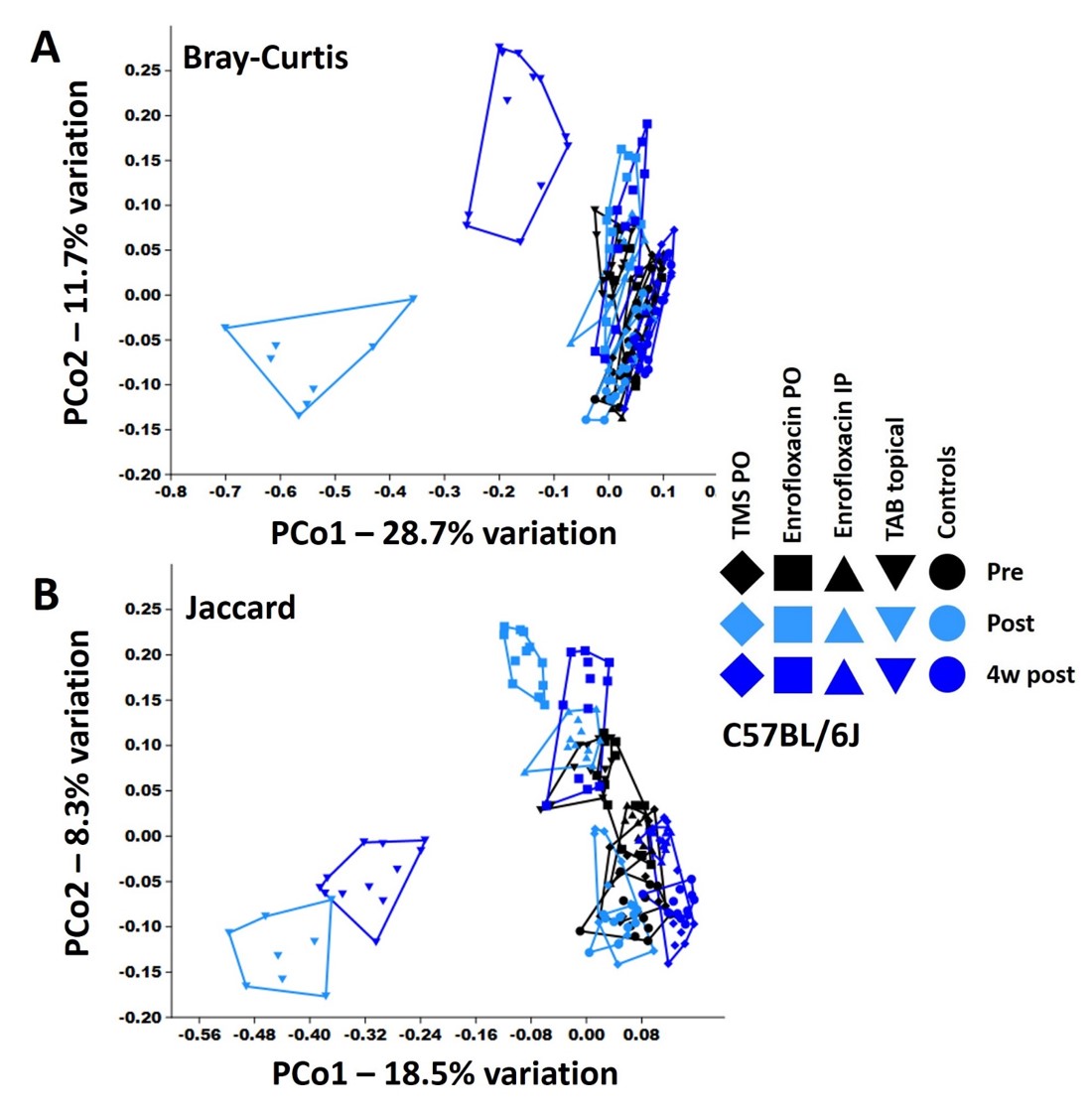

Supplement: Supplementary file 5 — Additional file 5. Principal coordinate analysis of samples collected from C57BL/6J mice (n = 12) before (pre), immediately after (post), and 4 weeks after (4w post) treatment with TMS PO, enrofloxacin PO, enrofloxacin IP, topical triple antibiotic (TAB), or no treatment (Control), ordinated using Bray-Curtis (A) or Jaccard (B) similarities. [file 13567_2020_839_MOESM5_ESM.jpg]
